# Preclinical evaluation of targeted therapies for central nervous system metastases

**DOI:** 10.1242/dmm.050836

**Published:** 2024-09-30

**Authors:** Alexander J. Pfeil, Joshua D. Hale, Tiger S. Zhang, Kentaro Wakayama, Isao Miyazaki, Igor Odintsov, Romel Somwar

**Affiliations:** ^1^ Department of Pathology and Laboratory Medicine, Memorial Sloan Kettering Cancer Center, New York, NY 10065, USA; ^2^ University of North Carolina School of Medicine, Chapel Hill, NC 27514, USA; ^3^ Taiho Pharmaceutical Co. Ltd. 3, Okubo, Tsukuba, Ibaraki 300-2611, Japan; ^4^ Department of Pathology, Brigham and Women's Hospital, Harvard Medical School, Boston, MA 021105, USA; ^5^ Human Oncology and Pathogenesis Program, Memorial Sloan Kettering Cancer Center, New York, NY 10065, USA

**Keywords:** Targeted therapy, Non-small cell lung cancer, Central nervous system metastasis, Blood–brain barrier

## Abstract

The central nervous system (CNS) represents a site of sanctuary for many metastatic tumors when systemic therapies that control the primary tumor cannot effectively penetrate intracranial lesions. Non-small cell lung cancers (NSCLCs) are the most likely of all neoplasms to metastasize to the brain, with up to 60% of patients developing CNS metastases during the disease process. Targeted therapies such as tyrosine kinase inhibitors (TKIs) have helped reduce lung cancer mortality but vary considerably in their capacity to control CNS metastases. The ability of these therapies to effectively target lesions in the CNS depends on several of their pharmacokinetic properties, including blood–brain barrier permeability, affinity for efflux transporters, and binding affinity for both plasma and brain tissue. Despite the existence of numerous preclinical models with which to characterize these properties, many targeted therapies have not been rigorously tested for CNS penetration during the discovery process, whereas some made it through preclinical testing despite poor brain penetration kinetics. Several TKIs have now been engineered with the characteristics of CNS-penetrant drugs, with clinical trials proving these efforts fruitful. This Review outlines the extent and variability of preclinical evidence for the efficacy of NSCLC-targeted therapies, which have been approved by the US Food and Drug Administration (FDA) or are in development, for treating CNS metastases, and how these data correlate with clinical outcomes.

## Teaser

**Summary:** This Review discusses the preclinical evidence for targeted therapies in treating central nervous system metastases and how these data correlate with clinical outcomes.

## Introduction

Metastatic brain tumors are a significant cause of morbidity and mortality for patients with cancer. Patients with solid tumors have an estimated 10-40% chance of developing metastasis to the brain during the disease course, although the incidence of brain metastases varies greatly among different tumor types (Aizer et al., 2022). For patients presenting with brain metastases at the time of diagnosis of their primary malignancy, prognosis and treatment will depend on the primary tumor type, performance status, disease extent and tumor-specific factors. Most of these patients will have a median overall survival (OS; see Glossary, Box 1) time of between 3 and 16 months (Lamba et al., 2021). Primary neoplasms that have the highest propensity to spread to the central nervous system (CNS) are lung, breast, melanoma, renal cell and colorectal cancers (Lamba et al., 2021; Sacks and Rahman, 2020).

Lung cancer is the third most common primary neoplasm and the most common cause of malignancy-related death in the USA (Leiter et al., 2023). This is due, in part, to the propensity of lung cancer to metastasize to the brain. About 16-20% of patients with non-small cell lung cancers (NSCLCs) will present with brain metastasis at diagnosis and 40-60% of patients will eventually develop brain metastases during the disease course (Lamba et al., 2021; Rangachari et al., 2015). Additionally, patients with NSCLCs are more likely than patients with other primary neoplasms to develop multiple metastases in the brain, contributing in part to their relatively worse prognosis and more complex treatment regimens (Sacks and Rahman, 2020). The median survival of patients with NSCLCs with brain metastasis is currently around 12 months, increasing to 15 months in those with adenocarcinoma as the primary tumor histology (Sperduto et al., 2020).

The treatment options for patients with NSCLCs with brain metastases is complex and often combines local (surgery and/or radiation) and systemic therapies. For those presenting with signs of impending herniation (Box 1) or with solitary, large intracranial lesions, neurosurgery or stereotactic radiosurgery (SRS; Box 1) are effective interventions with the potential for long-term survival (Lamba et al., 2019). For patients with multiple lesions, either whole-brain radiation therapy (WBRT) or SRS is considered. Previously, patients with multiple, small brain metastases received WBRT as a first-line therapy, but SRS is now the preferred option as it causes fewer and less severe toxicities than WBRT (Myall et al., 2021). Adjuvant therapies for WBRT and SRS include immunotherapy, chemotherapy and, for those with targetable driver mutations, small-molecule inhibitors, although these systemic therapies are more frequently being used as standalone treatments in eligible patients (Myall et al., 2021).

Advances in genome sequencing have enabled some of the most common driver mutations in NSCLC to be targeted using small-molecule inhibitors (Box 2), particularly tyrosine kinase inhibitors (TKIs). In the past two decades, these targeted therapies have transformed the treatment landscape of NSCLC, leading to a steady decline in mortality in patients with actionable driver mutations (Barr Kumarakulasinghe et al., 2015). TKIs are incorporated into the systemic therapy regimen for patients based on the specific mutational profile of their tumor and, increasingly, these therapies are being utilized in the frontline management of intracranial metastasis. However, the duration of activity of these drugs is limited by several factors: acquired tumor resistance mechanisms, such as binding site mutations; the activation of alternative signaling pathways; and metastasis to sanctuary sites, such as the brain, where the ability of the drugs to cross the blood–brain barrier (BBB) remains a significant challenge to effectively treating brain metastases (Meador and Hata, 2020). Hence, research aimed at recognizing and overcoming these mechanisms is crucial to maximizing the clinical utility of these therapeutics.

In this Review, the challenges and opportunities in developing and evaluating targeted therapies for treating brain metastases will be discussed. Specifically, the preclinical evidence for TKIs in treating NSCLC-derived brain metastases will be reviewed and correlated to clinical outcomes seen in clinical trials.

**Box 1. Glossary**

**Cassette dosing:** a technique used for efficiently assessing the pharmacokinetic profiles of drugs via dosing multiple compounds in a single model rather than dosing each compound individually.

**Extravasation:** the process by which cancer cells exit the circulatory system and invade surrounding tissues. Extravasation is a key step in the metastatic cascade.

**Herniation (brain):** a potentially life-threatening medical condition that occurs when increased intracranial pressure causes displacement of brain tissue from its normal position inside the skull.

**IC_50_:** a measurement used to indicate the concentration of a compound needed to inhibit a specific biological process by 50%.

**Intracranial disease control rate:** the percentage of patients whose intracranial tumors shrink or remain stable over a defined period in a clinical trial. It is the sum of the complete, partial and stable disease rates.

**Intracranial time to treatment progression (iTTP):** the length of time from the initiation of treatment until an intracranial tumor shows signs of progression, either through growth in size or new tumors in the brain.

**Overall survival:** the length of time from randomization in a clinical trial until death.

**Progression-free survival (PFS):** the length of time after treatment without worsening of disease via increase in tumor size, development of new metastases or death.

**Quantitative structure–activity relationship (QSAR) model:** mathematical models that predict the activity of a chemical compound based on its structure.

**Stereotactic injection:** injection of a drug or disease component directly into the target tissue of interest.

**Stereotactic radiosurgery (SRS):** a form of radiation therapy that delivers radiation at therapeutic doses directly to cancerous lesions using three-dimensional imaging to specify the radiation target. Used to limit radiation exposure to non-cancerous cells.

**Box 2. Targetable driver mutations in non-small cell lung cancer**

Mutations that activate receptor tyrosine kinases or their downstream effectors are common oncogenic drivers in non-small cell lung cancer. These mutations generate hyperactive proteins that amplify growth and survival signals emanating from signaling networks, such as the phosphoinositide 3-kinase (PI3K)/protein kinase B (AKT), mitogen-activated protein kinase (MAPK), Janus kinase (JAK)/signal transducer and activator of transcription (STAT), and mammalian target of rapamycin (mTOR) pathways, to promote tumorigenesis and migration (Daga et al., 2015). In non-small cell lung cancer, some of the most common oncogenic drivers include epidermal-like growth factor (EGFR), Kirsten rat sarcoma virus (KRAS), anaplastic lymphoma kinase (ALK), human epidermal growth factor 2 (HER2), c-ROS oncogene 1 (ROS1) and ‘rearranged during transfection’ protein (RET) (Chevallier et al., 2021).

## The BBB

**Fig. 1.**
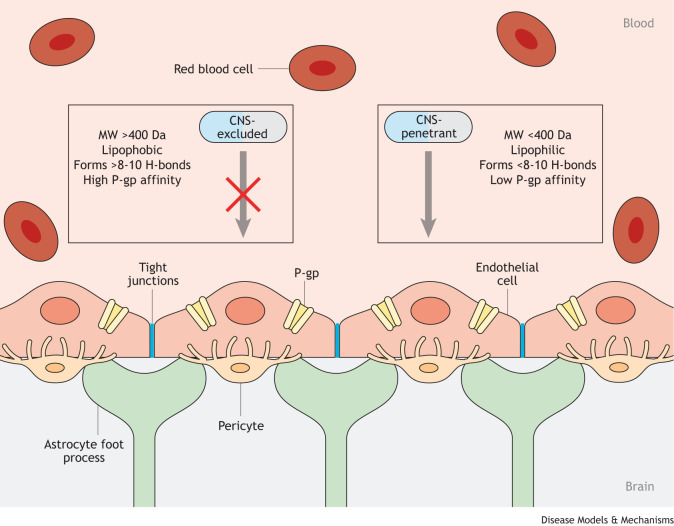
**The blood–brain barrier.** The blood–brain barrier (BBB) consists of a highly regulated neurovascular unit composed of endothelial cells connected by tight junctions, as well as pericytes and astrocytic foot processes. Together, these cellular components maintain central nervous system (CNS) homeostasis by tightly controlling intercellular and intracellular transport. Only therapies with chemical properties that enable them to permeate this barrier and be retained in the brain parenchyma can exert therapeutic efficacy. The drug properties most conducive to CNS-penetration include low molecular weight (MW) [<400 Dalton (Da)], high lipophilicity, less than eight to ten potential hydrogen-bonding sites, and low affinity for efflux transporters, including P-glycoprotein (P-gp) and breast cancer resistance protein (BCRP). The BBB may be damaged by infiltrating neoplastic lesions and/or local therapy (radiosurgery), although the significance of this damage to drug penetration is not fully understood.

The challenge of developing therapeutics for intracranial tumors primarily lies in penetrating the BBB. The BBB is created by specialized endothelial cells that are connected by tight junctions and surrounded by pericytes and astrocyte foot processes (Fig. 1). This neurovascular unit forms a unique barrier that limits the ability of drugs and disease to cross into the CNS. Neoplasms in the brain are known to compromise the BBB and, hence, also form a blood–tumor barrier (BTB), with heterogenous properties among the varying tumor types and grades (Arvanitis et al., 2020).

A key step in metastasis is extravasation (Box 1) of circulating tumor cells via interactions with cell adhesion molecules of the vasculature endothelium (Kienast et al., 2010). Tumor cell extravasation into the brain, and the subsequent colonization and proliferation of tumor cells in the extracellular matrix might damage endothelial cell tight junctions, creating a BTB that is ‘leaky’ relative to the BBB (Arvanitis et al., 2020). The extent and relevance of BBB or BTB breakdown, as caused by tumor cell infiltration, is not fully understood and likely exists as a spectrum in which larger tumors and, specifically, the centers of these tumors are less protected from systemic drug exposure relative to smaller lesions and those on the actively dividing invasive edge (Deeken and LöScher, 2007; Muldoon et al., 2007). Magnetic resonance imaging (MRI) and positron emission tomography (PET) have identified disruption of the BBB with human tumors, particularly with high-grade brain tumors (Sarkaria et al., 2018). Using murine models of brain metastasis of breast cancer, Lockman et al. (2010) have shown that 89% of brain metastases had partial BBB permeability, but that the uptake and efficacy of systemic chemotherapies were still greatly diminished by the residual BBB function. Given these data and the reduced efficacy of most systemic therapies in treating intracranial lesions, it remains vitally important to develop drugs that can penetrate a functioning BBB rather than depend on the select few lesions that might be vulnerable to systemic exposures.

The ability of compounds to permeate the BBB depends on several factors, including their molecular weight, lipophilicity, number of hydrogen bond donors and affinity for efflux transporters, which are major barriers to successful drug penetrance and retention (Wrobel and Toborek, 2016; Pandit et al., 2020). Smaller (<400 Da), non-polar molecules with low affinity for efflux transporters have the greatest intracranial penetrance owing to their ability to navigate the BBB (Pardridge, 2003) (Fig. 1). The major efflux transporters that have been linked to decreasing drug efficacy are P-glycoprotein (P-gp, also known as ABCB1) and breast cancer resistance protein (BCRP, also known as ABCG2) of the ATP-binding cassette gene family, with P-gp being most commonly implicated in resistance to therapy (Wanek et al., 2015; Lemos et al., 2008; Zhang et al., 2015). The characterization of these efflux transporters has led to the development of TKIs that are specifically designed to have low affinity for P-gp to increase their intracranial efficacy (Johnson et al., 2014).

The combination of these unique physiological and pharmacokinetic factors has made targeting brain metastases especially difficult. As a result, the creation of effective preclinical models for identifying CNS-active therapeutics has become increasingly important in drug discovery.

## Preclinical assessment of CNS penetration and metastasis control

In addition to its specific target engagement and pharmacodynamic properties, the capacity of a drug to effectively target CNS lesions depends on its ability to penetrate the BBB and be retained in the CNS (Pardridge, 2005). As a result, newer-generation TKIs are being specifically engineered to optimize these variables while retaining appropriately low IC_50_ values (Box 1) against the targeted protein (Zeng et al., 2015a; Johnson et al., 2014; Kettle et al., 2023). This drug development process can now be initiated using *in silico* models, such as quantitative structure–activity relationship (QSAR) models (Box 1), which can predict the pharmacokinetics of a drug and its potential for brain exposure based on its structural characteristics (Varadharajan et al., 2015; Fridén et al., 2009b). Following initial *in silico* modeling, rigorous *in vitro* and *in vivo* testing for efflux substrate affinity and for retention of the concentration of the unbound drug in the CNS must be done in order to assess the clinical utility of a compound.

### Animal models for measuring intracranial drug response

Rodents are the most commonly used preclinical animal models of metastasis and are frequently utilized to demonstrate the efficacy of a drug against intracranial tumors. These results are then often used to extrapolate the CNS-penetrant properties of the tested therapeutic. Although mice are tractable and practical to use as a preclinical *in vivo* cancer model, important anatomical and physiological differences exist between the mouse and human CNS. These differences have fueled interest in creating models to study brain tumors and CNS pharmacokinetics in other organisms (Hicks et al., 2021). Most notably, animal models of gliomas now include porcine (pig), canine (dog) and non-human primate subjects (Selek et al., 2014; Herranz et al., 2016; Rodgers et al., 2020). These larger models come with increased costs, care and handling needs, and ethical considerations, but more closely recapitulate intracranial drug penetration in humans owing to similarities in brain anatomy and greater homology of the P-gp transporter relative to those in rodents (Syvänen et al., 2009; Kido et al., 2022). As such, large animal models provide a potentially useful translational step between rodents and humans that may reduce the failure rate of promising drugs in clinical trials, although considerations of cost, time and ethics are expected to limit their utilization.

Once a drug has reached the preclinical animal model stage of development, the next consideration is the method of tumor initiation. Spontaneous models of metastasis are rarely used in drug design studies owing to the time required for brain metastases to become evident and the high likelihood of extracranial involvement, which lower throughput relative to that in other experimental models. In experimental metastasis models, CNS metastases are induced by tumor cell injection into the arterial circulation or intracranially into the brain (Daphu et al., 2013). Although tail vein injections are a common model for other sites of metastasis, such injections inefficiently seed the CNS, as tumor cells must first metastasize to the lung before entering the arterial circulation and spreading to the brain and other organs (Daphu et al., 2013; Francia et al., 2011).

Arterial circulation injections are done either by an intracardiac injection into the left ventricle or an intracarotid injection into the carotid artery. Intracardiac injection results in the spread of tumor cells throughout the systemic circulation and can lead to metastases in the brain and other organs (Daphu et al., 2013). When performing intracardiac injections, care must be taken to avoid damaging the ventricular wall, and precise control over the number of tumor cells injected can be challenging. In comparison, intracarotid injection leads to more specific and reproducible generation of brain metastases (Daphu et al., 2013; Lowery and Yu, 2017). Intracarotid injection can also be done with subsequent ligation of the injected artery, reducing the spread of injected cells to extracranial sites (Daphu et al., 2013). However, intracarotid injections can be more time and labor intensive, requiring a more difficult microsurgery to dissect out the carotid artery and imposing more risks to the animal (Lowery and Yu, 2017).

Intracranial injections can also be used to consistently generate CNS tumors, usually via stereotactic injection (Box 1) (Boire et al., 2020; Miarka and Valiente, 2021; Daphu et al., 2013). Direct implantation of tumor cells into the brain is more appropriately referred to as a heterotopic model of local growth rather than a metastatic model, as the cells only face the final step of the metastatic cascade (Daphu et al., 2013). In contrast to intracardiac or intracarotid injections, intracranial injections allow for more accurate control over the number of cells injected and the location of the tumor in the brain, enabling the opposite brain hemisphere to be used as an internal control (Miarka and Valiente, 2021). In the intracranial injection model, tumor cells are implanted all at once and begin to expand immediately, offering a relatively long timespan in which to evaluate experimental therapeutics (Miarka and Valiente, 2021). However, the intracranial injection model has potential drawbacks relating to the injection method that need to be considered when interpreting studies of drug efficacy. As intracranial injections fail to replicate most of the steps of metastasis, including invasion of tumor cells through the BBB and initial colonization of the brain tissue, the local brain environment is likely to be very different than that of the metastatic niche in humans (Miarka and Valiente, 2021; Masmudi-Martín et al., 2021). In addition, the injection itself induces neuroinflammation, which may affect the BBB and penetration of experimental therapeutics (Miarka and Valiente, 2021; Wyatt and Davis, 2019).

The extent to which the method of tumor cell injection affects the penetration of experimental therapeutics and the response of metastases to therapy is not entirely clear. In a mouse model of HER2 (also known as ERBB2)-positive breast cancer CNS metastases, intracranial injection of tumor cells resulted in a more leaky BBB compared to intracardiac injection (Wyatt and Davis, 2019). Only a CNS-targeted nanoparticle was able to penetrate the BBB in the intracardiac injection model, whereas in the intracranial injection model, non-specific nanoparticles and a small-molecule drug were also able to penetrate the BBB. In another mouse model of CNS metastasis, generated from *MET* (encoding a receptor tyrosine kinase)-driven NSCLC, the intracranial injection of patient-derived xenografts resulted in variable increases in BBB leakiness (Friese-Hamim et al., 2022). All the implanted tumors had regions of disturbed and intact BBB, with between 20 and 77% of the tumor showing vascular permeability as measured by gadolinium contrast-enhanced T1-weighted MRI. However, the degree of vascular permeability did not correlate with the response to tepotinib, a MET inhibitor with good CNS penetration. It should be noted that most studies of CNS metastases using intracardiac, intracarotid or intracranial injection methods do not explicitly assess BBB vascular permeability via contrast-enhanced MRI but instead monitor tumor growth via bioluminescence or other imaging methods (Baschnagel et al., 2021; Chen et al., 2013; Kodack et al., 2012; Kodama et al., 2014; Yang et al., 2016; Zhang et al., 2021). Hence, we believe that intracranial injection models should not be used in isolation to determine the CNS-penetrant properties of a drug and should instead be coupled with pharmacokinetic studies on rodents with a non-manipulated BBB.

### Characterizing CNS penetration

Preclinical characterization of CNS penetration traditionally centered around comparing the concentration ratio of total drug in brain tissue homogenate with that in plasma samples, following drug administration to rodents. This measure is frequently called the partition coefficient *K*_p,brain_, or its logarithm, called logBB, is used. Although total drug concentration measurements from brain tissue homogenate and plasma are relatively straightforward to measure, *K*_p,brain_ does not accurately reflect the concentration of drug in a suitable state for therapeutic efficacy (Heffron, 2018; Clark, 2003). As such, logBB values can differ between CNS-active drugs by up to 2000-fold, highlighting their lack of predictive value in identifying successful drug candidates (Hammarlund-Udenaes et al., 2008). The free-drug hypothesis posits that only the concentration of a drug not bound to proteins or lipids can interact with its target and exert therapeutic effect (Summerfield et al., 2022). Hence, the partition coefficient must be corrected for the binding of the drug to the various components of brain tissue, as well as for protein binding in plasma. A more useful measurement of CNS partitioning than logBB values, therefore, is the unbound brain-to-plasma partition coefficient, *K*_p,uu,brain_ (Box 3).

**Box 3. Equations for characterizing central nervous system penetration**

The unbound brain-to-plasma partition coefficient *K*_p,uu,brain_ is defined by the equation:

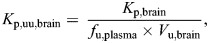

where *K*_p,brain_ is the concentration ratio of total drug in brain to that in plasma samples, *f*_u,plasma_ is the unbound drug fraction in plasma and *V*_u,brain_ is the unbound volume of distribution in brain tissue. *V*_u,brain_ represents the ratio of total drug concentration in the brain to the unbound concentration in brain interstitial fluid, which can be determined by *in vivo* microdialysis to measure drug concentrations or, more commonly, via *in vitro* uptake studies in brain slices (Neumaier et al., 2021). A common alternative to *V*_u,brain_ is the unbound drug fraction *f*_u,brain,_ which can be determined via equilibrium dialysis with brain tissue homogenate (Fridén et al., 2009a; Wan and Bergström, 2007; Varadharajan et al., 2015).

Efflux ratio (ER) is defined by the equation:

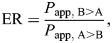

where *P*_app_ represents the apparent permeability coefficient across the cell monolayer, and B>A and A>B represent basolateral to apical and apical to basolateral directions of transport, respectively (Feng et al., 2008).

Compounds with a *K*_p,uu,brain_ of >0.3 in rodents are generally considered to have a high degree of free CNS penetration (Zhang et al., 2016; Heffron, 2016). Although *K*_p,uu,brain_ values <0.3 do not necessarily indicate a lack of CNS therapeutic efficacy, very low *K*_p,uu,brain_ values do indicate a need for a corresponding increase in systemic dosing, which often crosses the threshold of tolerability. Such a strategy of increasing dosage to overcome limited CNS penetration has been attempted with multiple TKIs, including those that target the epidermal growth factor receptor (EGFR). A retrospective analysis of 35 patients with NSCLC who underwent treatment with high-dose erlotinib to target mutant EGFR found that the high-dose treatment was efficacious and safe in some patients with refractory brain metastases (Kawamura et al., 2015). Although such strategies have been demonstrated in a small number of patients, newer-generation TKIs with significantly greater intracranial penetration and response rates offer a much safer treatment option.

**Fig. 2.**
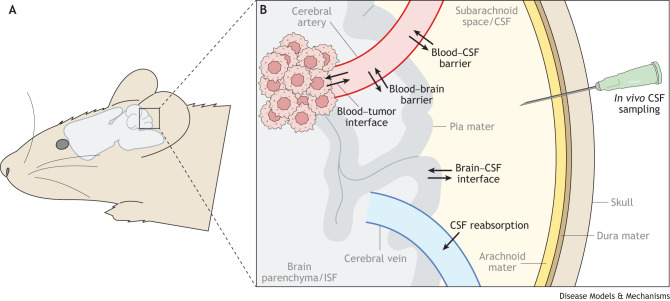
**Relationship between central nervous system compartments and vasculature.** (A) Cerebrospinal fluid (CSF) is most commonly sampled through the cisterna magna in mice (outlined by the small square). (B) CSF flows in the subarachnoid space of the vertebral column and skull, providing a dynamic circulatory system that maintains central nervous system (CNS) homeostasis before draining into the venous system. The systemic vasculature interfaces with the CSF in the subarachnoid space (blood–CSF barrier), the brain parenchyma (blood–brain barrier) and (in the context of neoplastic lesions) the periphery of a tumor (blood–tumor interface). Each of these interphases represent a unique physiological barrier, with differing degrees of permeability and efflux transporter abundance. To evaluate intracranial drug concentration, the CSF is often sampled from the subarachnoid space through the cisterna magna in mice (pictured here) or from the lumbar spine in humans. The CSF and interstitial fluid (ISF) of brain parenchyma are separated by the brain–CSF interface, which is primarily made up of ependymal cells and discontinuous gap junctions. Bulk flow of the ISF and solutes favors movement from the ISF to the CSF, and hence drug concentration in the CSF is generally concordant with that in the ISF.

Another common method used to approximate the unbound drug fraction in the brain, as well as to characterize CNS penetration more generally, is to measure drug concentrations in the cerebrospinal fluid (CSF) (*C*_CSF_), which is often expressed as a ratio relative to total or unbound plasma concentration (*K*_p,CSF_ or *K*_p,uu,CSF_). *C*_CSF_ is widely used as a proxy for the unbound drug concentration in brain interstitial fluid (ISF), and hence as the unbound drug concentration in brain tissue for *K*_p,uu,brain_ calculations, under the assumption that there is rapid equilibrium between the two compartments (Lin, 2008) (Fig. 2). *C*_CSF_ is much simpler to assess than the unbound drug concentration in the brain ISF in both preclinical models and clinical settings. CSF can be readily sampled from the subarachnoid space via a catheter into the cisternal magna in animals (Fig. 2) and via a lumbar spine puncture in humans, making it the most practical measurement of CNS penetrance clinically (de Lange, 2013). However, unbound drug equilibrium between the brain ISF and CSF compartments is not absolute and is complicated by the brain–CSF interface and by differences between the blood–CSF barrier and the BBB, where efflux transporters are unequally distributed (Shen et al., 2004). For drugs that are high-affinity substrates for P-gp, for example, *C*_CSF_ may overestimate the unbound drug concentration in the brain ISF (Kodaira et al., 2011; Venkatakrishnan et al., 2007). Generally, however, *C*_CSF_ has proven to be a reliable surrogate for predicting unbound drug concentration in the brain to assess brain penetration of the drug (Liu et al., 2009; Venkatakrishnan et al., 2007). It is also important to note that drug concentrations in human CSF tend to be higher and more variable than those observed in rats. This might be because most CSF measurements in humans occur under diseased conditions, although other factors such as species-specific efflux transporter dynamics and site of CSF withdrawal might also be at play (de Lange, 2013; Kanellakopoulou et al., 2008; Syvänen et al., 2009).

An important requirement to maximizing *K*_p,uu,brain_ is to ensure that a drug resists active efflux by P-gp or BCRP, among other efflux transporters (Hammarlund-Udenaes et al., 1997). A variety of *in vitro* assays have been developed to evaluate compounds as P-gp or BCRP substrates, with the most common being transwell-based assays using polarized epithelial cell lines that form tight junctions *in vitro*, such as the Madin–Darby canine kidney (MDCK) or LLC-PK1 cell lines (Gartzke and Fricker, 2014; Nicolaï et al., 2020). The MDCK or LLC-PK1 cell lines can be stably transfected with plasmids expressing human or mouse P-gp or BCRP and used to compare efflux ratios (Box 3) of select compounds between transfected and non-transfected controls. It is important to clarify whether mouse or human genes are used for expressing efflux transporters as certain drugs might have a different affinity for murine versus human homologs. Such a discrepancy was seen the mouse *Abcg2* and human *ABCG2* gene orthologs (encoding BCRP) in a study of the KRAS^G12C^ inhibitor sotorasib, as sotorasib had a high affinity for mouse *Abcg2* but not for human *ABCG2* (Loos et al., 2022). Caco-2, an intestinal epithelial cell line derived from human colon adenocarcinoma, also provides an acceptable model of BBB penetration for passive diffusion and affinity of efflux transport, especially if treated with vinblastine, which increases P-gp expression (Sun et al., 2008; Laska et al., 2002).

Drug candidates with a low efflux ratio in *in vitro* cell lines that overexpress P-gp and/or BCRP are considered to have a higher likelihood of achieving pharmacologically relevant *K*_p,uu,brain_ values. In addition, certain compounds that are substrates for these transporters can exert concentration-dependent inhibition of their own efflux, as was seen with the KRAS^G12C^-targeted therapy adagrasib (Sabari et al., 2022b). If this inhibition occurs in a physiologically relevant concentration range, the compound will likely bypass efflux and gain access to the CNS. Although these systems represent an attractive way to initially evaluate passive diffusion properties and transporter efflux, structural differences between epithelial and endothelial cells and a lack of a broader BBB microenvironment are important limitations to consider when extrapolating *in vitro* results to *in vivo* models.

## Targeted therapies in the treatment of NSCLC brain metastasis

Relative to most systemic therapies, such as chemotherapies or monoclonal antibodies, small-molecule TKIs have low molecular weights and are hypothetically more amenable to simple diffusion across the BBB. However, many TKIs achieve poor levels of CNS penetration, with CSF concentrations often <5% of those seen in circulating plasma (Togashi et al., 2012; Costa et al., 2011). The primary barrier for many of these compounds is efflux transporter proteins, which have a high affinity for different classes of TKIs, including most early-generation EGFR, anaplastic lymphoma kinase (ALK) and RET inhibitors (Table 1).

**Table 1.**
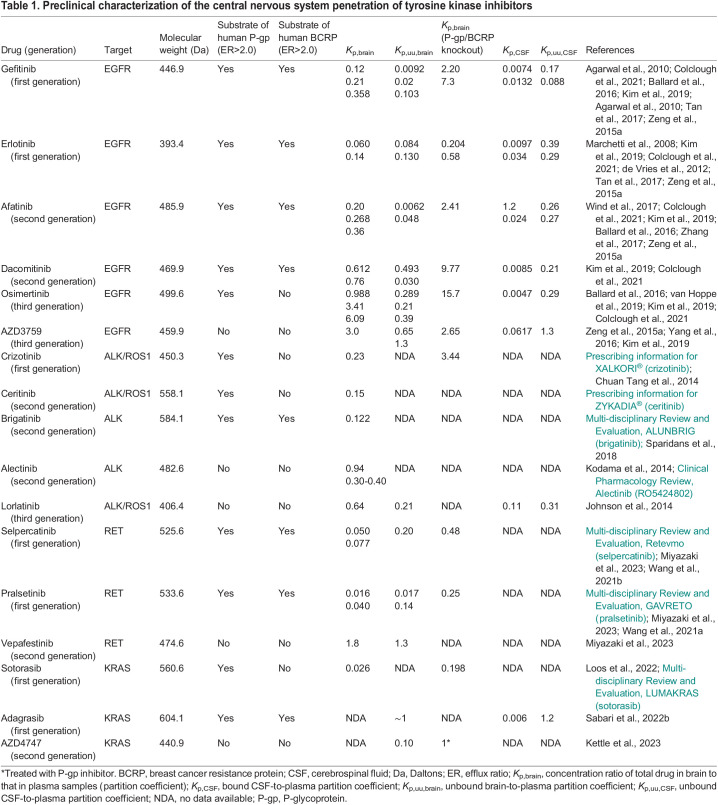
Preclinical characterization of the central nervous system penetration of tyrosine kinase inhibitors

Despite the general lack of preclinical data on TKI penetration into the CNS, TKIs have shown modest efficacy in controlling CNS metastases, producing promising outcomes in preselected patient populations that possess targetable mutations. Relative to intracranial overall response rates (iORRs) of 30-60% for WBRT and 30-40% for chemotherapy, small-molecule TKIs regularly have response rates of up 70-80% in patients with NSCLC with targetable mutations (Khan and Dicker, 2013; Barlesi et al., 2011; Park et al., 2012; Porta et al., 2011; Wu et al., 2013; Antoniou et al., 2005; Cortes et al., 2002). Intracranial tumor relapse following TKI treatment is still of concern, although the rate of CNS progression in patients with NSCLCs with *EGFR* mutations is lower in those given first-line TKIs (6% of whom have a 12-month risk of CNS progression) versus in those given chemotherapy (19% of whom have a 12-month risk of CNS progression) (Heon et al., 2012). *ALK*-rearranged disease, which has an especially high propensity for CNS metastasis, has also seen promising results with targeted inhibitors despite a general lack of preclinical evidence for CNS penetration (Table 1). Thus, despite limited preclinical evidence of CNS penetration, targeted therapies in driver mutation-selected patient populations are increasingly recognized as being clinically superior to radiation therapy or other systemic therapies for first line treatment of NSCLC brain metastases. It has been hypothesized that concurrent radiation to the brain could temporarily damage the BBB, enabling TKIs to better penetrate the brain and produce superior outcomes (Zeng et al., 2015b). However, it is still to be determined whether radiation therapy alongside TKIs further improves outcomes, with mixed results seen thus far (Jiang et al., 2016; Wang et al., 2018).

**Fig. 3.**
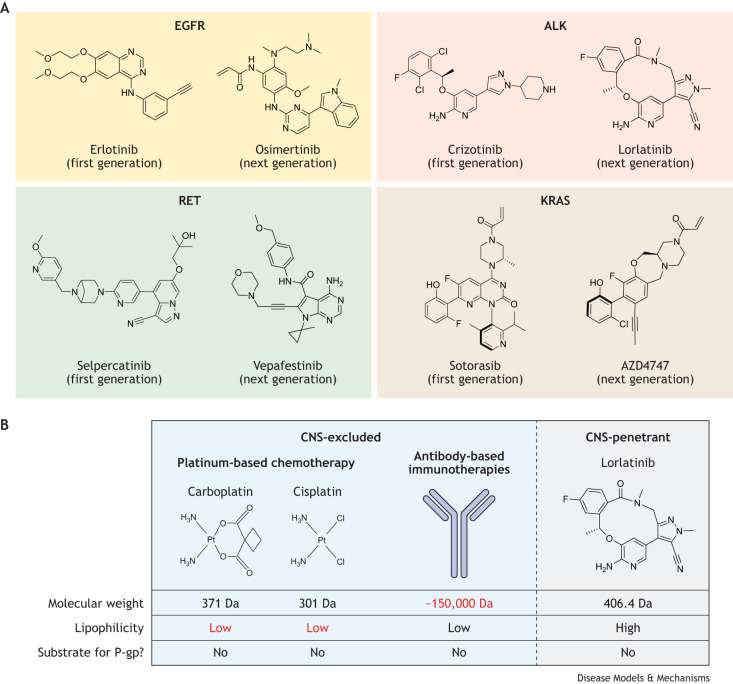
**First-in-class versus next-generation tyrosine kinase inhibitors.** (A) In comparison to chemotherapy, antibody-based therapeutics or radiation, small-molecule tyrosine kinase inhibitors (TKIs) demonstrate greater efficacy against brain metastases. However, most first-in-class TKIs have poor penetration into the central nervous system (CNS). Newer-generation TKIs have been specifically engineered to possess the pharmacokinetic properties necessary for treating intracranial disease. This has been achieved through optimizing parameters such as molecular weight, polar surface area and low affinity for efflux transporters, while retaining a high degree of kinase selectivity against their respective targets. Some examples of the chemical structures of both first- and next-generation TKIs are shown here. (B) Other systemic therapies can suffer from poor CNS penetration owing to multiple factors including size limitations (antibody-based therapeutics) or lipophobic properties (platinum-based chemotherapy). The properties of lorlatinib, as an example of a CNS-penetrant TKI, are shown. ALK, anaplastic lymphoma kinase; Da, Dalton; EGFR, epidermal growth factor receptor; KRAS, Kirsten rat sarcoma virus; P-gp, P-glycoprotein; RET, ‘rearranged during transfection’ protein.

Newer-generation TKIs, such as osimertinib (which selectively targets EGFR with specific kinase domain mutations) and lorlatinib (which targets ALK) have been developed specifically to better penetrate the CNS and produce improved outcomes in advanced NSCLC over their early-generation counterparts (Nelson and Wang, 2023; Thomas et al., 2022) (Fig. 3). Likewise, vepafestinib, a novel inhibitor of RET, has been developed with pharmacokinetic properties that improve its CNS penetration, relative to the two US Food and Drug Administration (FDA)-approved RET inhibitors selpercatinib and pralsetinib (Miyazaki et al., 2023), both of which already show impressive intracranial activity with iORRs of around 70-80% (Drilon et al., 2019; Gainor et al., 2019; Subbiah et al., 2021; Griesinger et al., 2022). The intracranial activity of the novel class of drugs that target KRAS^G12C^, namely, sotorasib and adagrasib, was not initially explored in published preclinical investigations, but recent analyses have suggested that they both have modest activity (Loos et al., 2022; Sabari et al., 2022b).

In the following sections, we discuss the preclinical findings from each class of these drugs in terms of their CNS penetration and preclinical CNS efficacy, and how these measures correlate with clinical outcomes.

### EGFR

The most common targetable mutations in NSCLC are found within *EGFR*. About 20-76% of patients with NSCLC (with ethnicity accounting for this broad range) possess an *EGFR* mutation and there are many effective small-molecule inhibitors targeting this receptor tyrosine kinase (Midha et al., 2015; Aizer et al., 2022). Patients with *EGFR*-mutant NSCLC have a higher incidence of brain metastases relative to those with wild-type *EGFR* (Hsu et al., 2016; Li et al., 2016). The first-generation EGFR inhibitors, gefitinib and erlotinib, were the first TKIs approved by the FDA for use in NSCLC, with gefitinib approved in 2003 and erlotinib in 2004. These first-generation inhibitors demonstrate poor preclinical CNS penetration, as both have a strong affinity for efflux transporters and demonstrate low levels in the CSF and low free brain penetration levels in rodent models (Table 1). The second-generation EGFR inhibitors, afatinib and dacomitinib, have similar preclinical findings, although one study that used cassette dosing (Box 1) found dacomitinib to have a *K*_p,uu,brain_ value of >0.3, indicating acceptable levels of CNS penetration (Kim et al., 2019). However, another study utilizing single-compound dosing reported a *K*_p,uu,brain_ value for dacomitinib of 0.030 (Ballard et al., 2016), highlighting the potential for such measures to differ by 10-fold or more, depending on the study methodology. In humans, the CSF penetration rates of these first-generation EGFR-targeted therapies is also low. The highest penetration rate reported for erlotinib is 2.15-5.1% and 1-3% for gefitinib (Chen et al., 2023; Fang et al., 2015; Togashi et al., 2012).

Despite these therapies being developed without CNS penetration in mind, they show clinical efficacy that is greater than that expected given their preclinical CNS penetration parameters alone (Table 2). An analysis of 16 NSCLC studies found a pooled median progression-free survival (PFS; Box 1) of 7.4 months (95% c.i., 4.9-9.9 months) and overall survival of 11.9 months (95% c.i., 7.7-16.2 months) in unselected patients with brain metastases treated with first-generation EGFR TKIs. Furthermore, they found longer PFS (12.3 months versus 5.9 months) and overall survival (16.2 months versus 10.3 months) in patients with *EGFR* mutations versus those in the unselected group (Fan et al., 2014). Afatinib has also demonstrated intracranial activity. A retrospective analysis of 74 treatment-naïve patients with advanced *EGFR*-mutant NSCLC and brain metastases treated with afatinib reported an iORR of 81.1% (Wei et al., 2019). In addition, an exploratory combined analysis of the LUX-Lung 3 and LUX-Lung 6 clinical trials found that afatinib improved PFS in patients with *EGFR*-mutant NSCLC and brain metastases, relative to chemotherapy (8.2 months versus 5.4 months, *P*=0.0297) (Schuler et al., 2016). Likewise, a recent phase II trial of dacomitinib in 30 patients with NSCLC brain metastasis reported a remarkable 96.7% iORR and an intracranial complete response in 63.3% of patients (Jung et al., 2023).

**Table 2.**
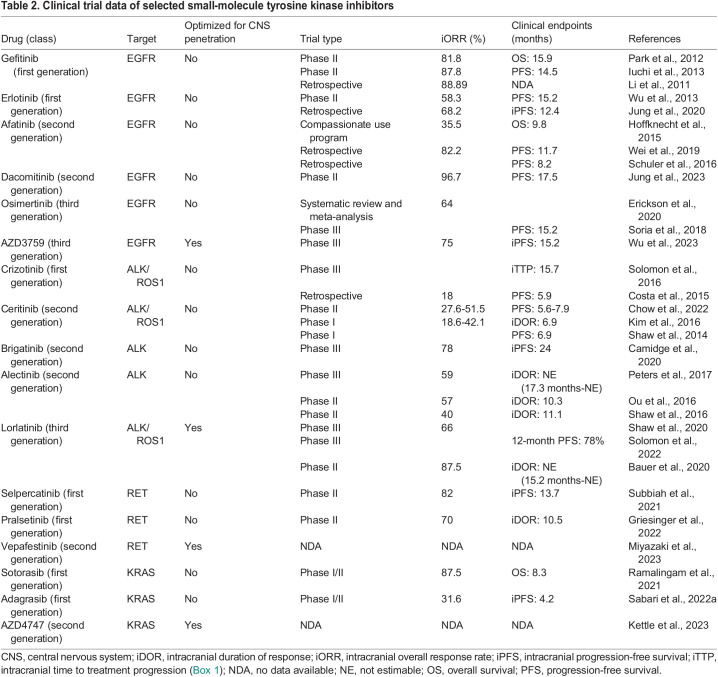
Clinical trial data of selected small-molecule tyrosine kinase inhibitors

Osimertinib is a third-generation EGFR TKI selective for exon 19 deletions, exon 21 L858R mutations, and the first-generation EGFR TKI-resistance mutation T790M. This drug possesses the highest average *K*_p,uu,brain_ value of FDA-approved EGFR inhibitors with a reported range of 0.21-0.39 (Table 1). Despite the above average *K*_p,uu,brain_ value, osimertinib is a substrate for P-gp and possesses *K*_p,uu,CSF_ values in rodents comparable to those of earlier-generation EGFR TKIs (Table 1). However, osimertinib has been shown to accumulate in non-human primate brains (as measured with PET imaging) as well as in human CSF, with a reported CSF-to-free plasma ratio of ∼16% (Yang et al., 2020; Ballard et al., 2016). These improved measures of CNS penetration appear to have improved outcomes in clinical trials of patients with *EGFR*-mutant NSCLC with brain metastasis treated with osimertinib compared to outcomes in those treated with early-generation EGFR TKIs. A phase III trial that compared osimertinib directly to first-generation EGFR TKIs found improved median PFS in all predefined patient subgroups, including those with CNS metastases (15.2 months versus 9.6 months, *P*<0.001) (Soria et al., 2018). The impressive intracranial efficacy of osimertinib has promoted its use as the recommended treatment for asymptomatic brain metastases that arise from *EGFR*-mutant NSCLC (Vogelbaum et al., 2022).

AZD3759 is an EGFR-targeted TKI that has been specifically developed for brain penetration and is currently under clinical investigation (Zeng et al., 2015a). AZD3759 is not a substrate for P-gp or BCRP and possesses *K*_p,uu,brain_ and *K*_p,uu,CSF_ values greater than 1, indicating similar unbound exposure in the plasma and brain (Table 1). AZD3759 shows impressive CNS penetration in humans, with unbound CSF-to-plasma values of 111% (Ahn et al., 2017). A phase III clinical trial has demonstrated its clinical superiority to gefitinib or erlotinib, with median intracranial PFS duration of 15.2 months versus 8.3 months (*P*<0.0001) and overall PFS of 9.6 months versus 6.9 months in *EGFR*-mutant NSCLC with CNS metastasis (*P*=0.0024) (Wu et al., 2023).

These results described above show that although early-generation EGFR TKIs are clinically efficacious in treating brain metastases in patients with *EGFR* mutations, the improved pharmacokinetic properties of the more recently developed TKIs, such as osimertinib and AZD3759, increase the CNS penetration of the molecules and thus offer significant improvements in controlling intracranial disease.

### ALK

ALK was initially characterized in 1994 as part of the nucleophosmin (NPM1)-ALK fusion protein expressed in the majority of anaplastic large-cell lymphomas (Morris et al., 1994; Roskoski, 2013). Physiological ALK is believed to play a role in nervous system development, whereas its constitutive activation in fusion proteins leads to activation of oncogenic pathways such as the mitogen-activated protein kinase (MAPK), Janus kinase (JAK)/signal transducer and activator of transcription (STAT) and the phosphoinositide 3-kinase (PI3K)/protein kinase B (AKT) pathways (Palmer et al., 2009). Approximately 5% of NSCLCs are associated with *ALK* chromosomal rearrangement, with echinoderm microtubule-associated protein-like 4 (EML4) being the most common fusion partner (Shreenivas et al., 2023; Devarakonda et al., 2015). Patients with ALK fusions are at a higher risk of brain metastasis relative to those with *EGFR* or other driver mutations (Kang et al., 2014; Fallet et al., 2014). As with early-generation EGFR-targeted TKIs, early-generation ALK inhibitors were not developed to penetrate the brain and hence have limited preclinical evidence as to their CNS-penetrant properties (Table 1). The first-generation ALK inhibitor, crizotinib, is a substrate for P-gp and demonstrates exceedingly low CSF penetration in humans, ranging from 0.06 to 0.26% (Costa et al., 2011; Metro et al., 2015). The clinical outcome data for crizotinib in treating NSCLC brain metastases also show its limited efficacy (Table 2).

The second-generation ALK inhibitors, ceritinib, alectinib and brigatinib, generally show higher CNS penetration in humans, with reported CSF-to-unbound plasma concentration ratios of 13-35% for ceritinib and 86% for alectinib (Chow et al., 2022; Gadgeel et al., 2014). Based on the limited preclinical literature, alectinib is predicted to have the greatest intracranial efficacy of the first- or second-generation ALK inhibitors as it is not a substrate for either P-gp or BCRP and possesses a *K*_p,brain_ value of >0.30 (Table 1). A pooled analysis that included 15 clinical trials of first- and second-generation ALK inhibitors supports the intracranial efficacy of alectinib, reporting a pooled iORR of 48% (95% c.i., 32-63%), ranging from 18% for crizotinib to 79% for alectinib (Zhang et al., 2019). Brigatinib has also been demonstrated to have clinical utility in the treatment of intracranial lesions, with the phase III ALTA-1L trial demonstrating brigatinib to be superior to crizotinib in TKI-naïve patients with NSCLC brain metastases, with an impressive intracranial PFS of 24.0 months (Camidge et al., 2020).

Lorlatinib is a third-generation ALK/ROS1 (Box 2) inhibitor that is designed specifically for brain penetration. It has a low affinity for P-gp or BCRP and shows moderate CNS penetration in rodents, with a *K*_p,uu,brain_ value of 0.21 (Johnson et al., 2014). As expected from these preclinical findings, lorlatinib readily penetrates the CNS in humans, with a mean ratio of CSF-to-free plasma concentration of 75% seen in four patients with advanced ALK-positive or ROS1-positive NSCLC (Shaw et al., 2017). Lorlatinib is effective against brain metastases in both treatment-naïve patients and those previously treated with an earlier-generation TKI, with multiple trials demonstrating an iORR of 63-66% (Solomon et al., 2018; Bauer et al., 2020; Shaw et al., 2020). A Bayesian network meta-analysis of ALK inhibitor trials in NSCLC found lorlatinib to be superior in regard to PFS relative to other first-line therapies in patients with brain metastases, although the reported overall response rates were similar for lorlatinib and the second-generation ALK inhibitors (Zhao et al., 2021).

Thus, these findings show that a concordance exists between preclinical and clinical CNS penetration data in humans for drugs, such as lorlatinib, that are specifically designed to penetrate the BBB and be retained in the CNS. Second-generation TKIs also demonstrate impressive clinical activity despite the relative lack of supportive preclinical data.

### RET

The ‘rearranged during transfection’ (RET) protein is a receptor tyrosine kinase involved in renal morphogenesis, neural development and spermatogonia stem cell maintenance (Li et al., 2019). When constitutively activated by rearrangements or point mutations, *RET* becomes an oncogenic driver for multiple tumor types, including thyroid and lung cancers (Kato et al., 2017). RET fusions, most commonly with kinesin family 5B (KIF5B) or coiled-coil domain-containing protein 6 (CCDC6), are driver mutations in an estimated 1-2% of patients with NSCLC (Kohno et al., 2012; Gainor and Shaw, 2013). Two FDA-approved RET-selective inhibitors, selpercatinib and pralsetinib, are currently available for treatment of RET fusion-positive NSCLC and show durable efficacy as a first-line therapy, as well as impressive intracranial efficacy (Cascetta et al., 2021). Although both inhibitors are substrates for P-gp and BCRP, selpercatinib has a modest *K*_p,uu,brain_ value of 0.20 and pralsetinib has a wide-ranging *K*_p,uu,brain_ value of 0.017-0.14 (Table 1). Pralsetinib has been measured in human CSF in two case reports of patients with NSCLC, yielding CSF-to-unbound plasma ratios of 24.6-26.6% (Zhao et al., 2022; De Jong et al., 2023). In subgroup analyses of patients with pretreated brain metastases from their respective trials, patients with measurable intracranial metastasis had iORRs of 82% and 70% for selpercatinib and pralsetinib, respectively (Griesinger et al., 2022; Subbiah et al., 2021). In all 80 patients with brain metastases at baseline in the LIBRETTO-001 trial of selpercatinib, the median intracranial PFS was 13.7 months. Despite not possessing optimal preclinical characteristics to indicate CNS penetration, both RET-selective inhibitors have shown impressive clinical efficacy for the treatment of brain metastases (Table 2).

Vepafestinib is a second-generation RET inhibitor with a distinct binding mode to RET and superior selectivity relative to that of selpercatinib or pralsetinib (Miyazaki et al., 2023). This RET inhibitor was developed to achieve efficacy against common on-target resistance mechanisms that are seen following treatment with RET-targeted TKIs, as well as for improved CNS penetration. Vepafestinib has excellent preclinical CNS penetration properties, as it is a weak substrate for P-gp and BCRP and has a *K*_p,uu,brain_ value of 1.3 (Table 1). Accordingly, vepafestinib produces better survival and tumor control than selpercatinib in an orthotopic NSCLC model of CNS metastasis (Miyazaki et al., 2023). Currently, there is an ongoing phase I/II trial to study the safety and efficacy of vepafestinib in RET-altered solid tumors (Table 2; NCT04683250).

### KRAS

The rat sarcoma (RAS) genes are some of the most frequently mutated and well-studied proto-oncogenes, with approximately 20-30% of all human cancers possessing mutations to one of the RAS family genes (Prior et al., 2020). As a central regulator of the MAPK pathway, constitutively activated RAS leads to uncontrolled cellular proliferation independent of extracellular growth factor signaling. Once considered ‘undruggable’, KRAS^G12C^ has recently joined the list of targetable NSCLC biomarkers, with numerous selective and pan-RAS inhibitors under development (Uprety and Adjei, 2020). Currently, two FDA-approved selective KRAS^G12C^ inhibitors are available: adagrasib (approved in December 2022), and the first-in-class sotorasib (approved in May 2021) (Dhillon, 2023). These inhibitors demonstrated similar efficacy in their respective phase II trials, with overall response rates of 42.9 and 37.1% and PFS of 6.5 and 6.8 months, respectively (Jänne et al., 2022; Skoulidis et al., 2021). The initial trials excluded patients with active, untreated brain metastasis, leaving a limited body of evidence to evaluate the intracranial efficacy of these inhibitors. However, the CodeBreak 100 trial of sotorasib included patients with resected brain metastases and those who had received radiation therapy prior to the trial. A post hoc analysis of a subset of these patients with evaluable brain metastases reported 14/16 (87.5%) patients with intracranial disease control (Ramalingam et al., 2021) (Table 2). Early reports from an expansion cohort of the KRYSTAL-1 trial for adagrasib, which enrolled patients with active brain metastasis, reported an iORR of 31.6% and intracranial disease control rate (Box 1) of 84.2% (Sabari et al., 2022a) (Table 2).

Sabari et al. (2022b) characterized the preclinical CNS penetrant properties of adagrasib and reported the unbound brain-to-unbound plasma and CSF concentrations to be near 1. The researchers also found that although adagrasib is a substrate for P-gp, the IC_50_ for P-gp inhibition was within a physiologically relevant range, theoretically allowing for adagrasib to bypass efflux. There are little preclinical data concerning the CNS-penetrant effects of sotorasib, although one study found it to be a strong substrate for P-gp, which would limit its concentration in the CNS (Loos et al., 2022). As such, the available preclinical evidence indicates adagrasib could be used in patients with brain metastasis, although prospective trials will be required to confirm its efficacy.

Researchers from AstraZeneca recently developed a new KRAS^G12C^-targeted candidate, AZD4747, which is specifically optimized for CNS penetration (Kettle et al., 2023). AZD4747 is a weak substrate for P-gp or BCRP in *in vitro* models, although co-dosing mice with AZD4747 and a P-gp inhibitor raised its *K*_p,uu,brain_ value from 0.1 to 1, indicating the potential for discordance between *in vitro* and *in vivo* efflux transporter effects. The researchers also measured *in vivo K*_p,uu,brain_ values in dogs and monkeys, which were found to be 0.7 and 1.6, respectively. These results highlight the importance of inter-species variation in the CNS penetration of TKIs, such as AZD4747, which might explain why TKIs in other classes exert impressive intracranial disease control despite failing to efficiently penetrate the CNS of rodents.

## Conclusions

Despite advancements in treatment options for NSCLC-derived brain metastases, including more specific and potent targeted therapies, this class of tumor remains one of the most pressing clinical challenges to address, due to its high frequency relative to that of other tumor types and the difficulty of finding therapies suitable for both CNS penetration and tumor elimination. Although certain clinical contexts, such as large intracranial lesions or concurrent radiotherapy, may increase the permeability of the BBB to systemic therapies, it remains vital to develop drugs that can penetrate a functioning BBB to achieve broad clinical efficacy.

Drug developers have begun to consider CNS penetration as a central goal in drug design for new targeted therapies following a promising pipeline of *in silico* modeling, *in vitro* assays and preclinical animal models, before implementation in clinical trials. There still exist many challenges to effective preclinical evaluation of CNS-penetrant therapies, which expand beyond the study of small-molecule TKIs in NSCLC-derived brain metastases. These include the importance of inter-species differences in efflux transporter dynamics and CNS pharmacokinetics, and how to minimize the discordance between varying models and clinical trial data. In addition, a deeper understanding of how the BBB in a disease state impacts drug permeability, which is especially pertinent to larger therapies such as antibody-based therapeutics, will better inform the therapeutic properties required for successful treatment of intracranial lesions.

Despite these challenges, the therapies highlighted in this Review that are optimized for CNS penetration show improved efficacy relative to that of earlier-generation counterparts. These developments have aided in the advancement of TKIs into first-line treatments for patients with targetable NSCLC-derived brain metastases, allowing many to avoid the morbidity associated with radiation therapy while improving survival. It is important to note, however, that even the therapies that were not specifically designed for CNS penetration might still have impressive intracranial activity, including dacomitinib in *EGFR* mutants, alectinib and brigatinib in ALK-rearranged disease, and selpercatinib in RET fusions. Thus, future clinical trials of newer-generation TKIs must compare novel candidates to the best-in-class inhibitors for CNS penetration, rather than the earliest-generation inhibitors with proven limitations in the treatment of intracranial disease. Given the reasonably high degree of discordance between preclinical CNS penetration data and clinical efficacy, properly controlled trials are essential to ensure the superiority of newly optimized drugs against their current standard-of-care counterparts.
